# Forced sustained swimming exercise at optimal speed enhances growth of juvenile yellowtail kingfish (*Seriola lalandi*)

**DOI:** 10.3389/fphys.2014.00506

**Published:** 2015-01-08

**Authors:** Arjan P. Palstra, Daan Mes, Kasper Kusters, Jonathan A. C. Roques, Gert Flik, Kees Kloet, Robbert J. W. Blonk

**Affiliations:** ^1^Institute for Marine Resources and Ecosystem Studies, Wageningen Aquaculture^†^, Wageningen University and Research CentreYerseke, Netherlands; ^2^Department of Animal physiology, Institute for Water and Wetland Research, Radboud University NijmegenNijmegen, Netherlands; ^3^Silt BVIJmuiden, Netherlands

**Keywords:** swimming exercise, growth, optimal swimming speed, feed conversion ratio, Doppler ultrasound imaging, aquaculture

## Abstract

Swimming exercise at optimal speed may optimize growth performance of yellowtail kingfish in a recirculating aquaculture system. Therefore, optimal swimming speeds (*U*_opt_ in m s^−1^ or body lengths s^−1^, BL s^−1^) were assessed and then applied to determine the effects of long-term forced and sustained swimming at *U*_opt_ on growth performance of juvenile yellowtail kingfish. *U*_opt_ was quantified in Blazka-type swim-tunnels for 145, 206, and 311 mm juveniles resulting in values of: (1) 0.70 m s^−1^ or 4.83 BL s^−1^, (2) 0.82 m s^−1^ or 3.25 BL s^−1^, and (3) 0.85 m s^−1^ or 2.73 BL s^−1^. Combined with literature data from larger fish, a relation of *U*_opt_ (BL s^−1^) = 234.07(BL)^−0.779^ (*R*^2^ = 0.9909) was established for this species. Yellowtail kingfish, either forced to perform sustained swimming exercise at an optimal speed of 2.46 BL s^−1^ (“swimmers”) or allowed to perform spontaneous activity at low water flow (“resters”) in a newly designed 3600 L oval flume (with flow created by an impeller driven by an electric motor), were then compared. At the start of the experiment, ten fish were sampled representing the initial condition. After 18 days, swimmers (*n* = 23) showed a 92% greater increase in BL and 46% greater increase in BW as compared to resters (*n* = 23). As both groups were fed equal rations, feed conversion ratio (FCR) for swimmers was 1.21 vs. 1.74 for resters. Doppler ultrasound imaging showed a statistically significant higher blood flow (31%) in the ventral aorta of swimmers vs. resters (44 ± 3 vs. 34 ± 3 mL min^−1^, respectively, under anesthesia). Thus, growth performance can be rapidly improved by optimal swimming, without larger feed investments.

## Introduction

Aquaculture is facing an increasing demand for sustainably produced fish. There is a great need to optimize the conditions for fish growth, but without compromising health and welfare. Currently, significant numbers of commercially produced species of fish suffer from impaired well-being and high mortality (e.g., Castro et al., 2011, 2013). This may, at least partly, be explained by the fact that fishes cannot display their normal swimming behavior due to high densities or low water flow. A promising natural, non-invasive and economical tool to enhance growth may be the induction of swimming exercise at optimal swimming speeds (Palstra and Planas, 2011). Evidence suggests that the health and welfare of swimming fish is improved, not compromised (Castro et al., 2013; reviewed by Huntingford and Kadri, 2013).

Sustained swimming exercise improves growth in several teleostean fishes (Jobling et al., 1993; Davison, 1997; Palstra and Planas, 2011; Davison and Herbert, 2013). Exercise enhanced growth performance in salmonids, such as brook trout *Salvelinus fontinalis* (Leon, 1986; East and Magnan, 1987), brown trout *Salmo trutta* (Davison and Goldspink, 1977; Bugeon et al., 2003), rainbow trout *Oncorhynchus mykiss* (Greer Walker and Emerson, 1978; Houlihan and Laurent, 1987), Arctic charr *Salvelinus alpinus* (Christiansen et al., 1989, 1992; Grünbaum et al., 2008), and Atlantic salmon *Salmo salar* (Totland et al., 1987; Jørgensen and Jobling, 1993; Castro et al., 2011). Growth stimulation by swimming exercise has also been reported for non-salmonid species like yellowtail kingfish *Seriola lalandi* (Brown et al., 2011); gilthead seabream *Sparus aurata* (Ibarz et al., 2011; Sánchez-Gurmaches et al., 2013), whiting *Merlangius merlangus* (Hammer, 1994), striped bass *Morone saxatilis* (Young and Cech, 1993, 1994), qingbo *Spinibarbus sinensis* (Li et al., 2013) and the Amazon species matrinxa *Brycon amazonicus* (Arbeláez-Rojas and Moraes, 2010) and pacu *Piaractus mesopotamicus* (da Silva Nunes et al., 2013); also, zebrafish *Danio rerio* grow better when forced to swim (Palstra et al., 2010). The skeletal muscle thereby undergoes morphometrical and biochemical changes in response to exercise (Johnston and Moon, 1980; Davison, 1997; Johnston, 1999; Bugeon et al., 2003; Martin and Johnston, 2005; Rasmussen et al., 2013). Exercise increases muscle transcriptional activity underlying these changes, in particular genes involved in muscle growth and developmental processes (Magnoni et al., 2013; Palstra et al., 2013). Exercise further enhances cardiac muscle growth and increases maximum cardiac output and hematocrit levels (rainbow trout; Farrell et al., 1990, 1991), all well-known adaptations to meet increased oxygen demand of tissues. Long-term sustained exercise lowers basal plasma cortisol levels in salmonids (rainbow trout: Woodward and Smith, 1985; Postlethwaite and McDonald, 1995, and Atlantic salmon: Boesgaard et al., 1993; Herbert et al., 2011) and striped bass (Young and Cech, 1993) and therefore cortisol may be a key player in exerting the exercise effects through its pivotal role in the control of metabolism and energy allocation (Mommsen et al., 1999).

Exercise-enhanced growth is optimal at a particular swimming speed where a maximum of energy is diverted to the skeletal muscles and where a minimum is lost due to other processes. The swimming speed for optimal growth is most likely near optimal swimming speeds (*U*_opt_) where the cost of transport (COT, energy spent on swimming over a certain distance) is lowest and the energetic efficiency highest (Palstra et al., 2010; reviewed by Davison, 1997; Palstra and Planas, 2011; Davison and Herbert, 2013). Importantly, *U*_opt_ reflects very well the swimming speed for optimal growth in a variety of salmonid species and *Seriola* sp (reviewed by Davison and Herbert, 2013). At speeds below optimum, energy expenditure may increasingly go to activities such as aggression (type II allostatic overload: McEwen and Wingfield, 2003) and, at speeds above optimal, swimming soon becomes unsustainable and stressful leading to oxygen debt and eventually causing fatigue (reviewed by Davison, 1997; type I allostatic overload: McEwen and Wingfield, 2003). At *U*_opt_, fish use the maximum of their energy for swimming and promoting the development of an aerobic phenotype. It may well be that cortisol, as a key player, warrants an optimal physiological stress condition (eustress) at *U*_opt_ which explains that also other beneficial effects may occur at this speed.

The carangid yellowtail kingfish (*S. lalandi* Valenciennes, 1883) is distributed circumglobally in subtropical seas, usually inhabiting deep pelagic waters (Nakada, 2002). Yellowtail kingfish swimming has a gross aerobic cost of transport comparable to that of swimming salmon or tuna species (Clark and Seymour, 2006). Migration capacity of *S. lalandi* is also similar: individuals were shown to travel over 2000 km from Australia to New Zealand (Gillanders et al., 2001). As a prized sushi and sashimi fish, *S. lalandi* is farmed in net pens in the USA, Chile, South-Africa, Japan and Australia, but also has excellent potential as viable new fish species for on-land culture in a recirculating aquaculture system (RAS; Abbink et al., 2012; Orellana et al., 2014; Blanco Garcia et al., 2014). So far, one study has investigated the effect of exercise on *S. lalandi* growth, showing 10% growth rate gain for fish of marketable size (1600 g) when exercised at 0.75 body lengths (BL) s^−1^ (Brown et al., 2011). This exercise was found optimal for growth stimulation of these fish when compared with 0, 1.5, and 2.25 BL s^−1^ (at 21.1 ± 0.03°C; 13L:11D; in ambient seawater in 13 m^3^ tanks). Clearly, the potential of implementing exercise to improve growth rate in *S. lalandi* is indicated.

The first objective of this study is to assess the swimming performance of juvenile yellowtail kingfish, specifically the changes in optimal swimming speed during juvenile development. We subjected three size classes of juveniles to swim performance tests in swim-tunnels equipped with respirometers. Secondly, we then applied the relation between size and *U*_opt_ to determine the effects of long-term forced and sustained swimming at *U*_opt_ on growth performance. For this purpose we subjected two groups to either a water flow forcing fish to swim at *U*_opt_, or to a low water flow that allows spontaneous activity. A newly designed 3600 L oval-shaped recirculating swim-flume was used to induce straight line swimming. The physiological consequences of exercise on growth were assessed by quantification of differences in size and blood flow. Importantly, both groups of fish were fed equal rations to avoid that feed intake would act as confounder in explaining anticipated physiological differences. In the light of earlier results (Brown et al., 2011), we hypothesize that forced sustained, straight line swimming exercise at *U*_opt_ will enhance growth performance of yellowtail kingfish and increase blood flow.

## Materials and methods

### Ethics

All experiments were performed in accordance with relevant guidelines and regulations. Protocols used complied with the current laws of the Netherlands and were approved by the Animal Experimental Committee (DEC) of the Wageningen UR in Lelystad (The Netherlands) under numbers 2012012 and 2013162.

### Swim performance tests and respirometry in swim-tunnels

#### Experimental fish and conditions

Juvenile yellowtail kingfish (*n* = 33; *BL* = 88 mm, *BW* = ~ 8 g) were obtained from the farm Silt BV (IJmuiden, the Netherlands). They were transported from the farm to the IMARES facilities in Yerseke by truck within 3 h. Fish were housed under similar conditions as in the hatchery: in natural seawater that was mixed with tap-water to 25.1 ± 0.1‰, at 23.4 ± 0.1°C and under a light regime of 16L:8D before and during the experimental periods. Fish were hand-fed three times per day at 7% BW day^−1^ with commercial feed (Skretting, Boxmeer, The Netherlands). Water quality was monitored daily for O_2_, pH, NH_4_, NO_2_, and NO_3_. Oxygen levels were 7.54 ± 0.08 mg L^−1^, pH was 8.07 ± 0.07, NH_4_-N averaged 0.27 ± 0.05 mg L^−1^, NO_2_-N averaged 1.5 ± 0.2 mg L^−1^ and NO_3_-N averaged 8.9 ± 0.9 mg L^−1^ over the 2.5 months experimental period. During three periods in time, subsamples of this batch of fish were used for swim performance tests (size groups 1–3; Table 1).

**Table 1:** **Size of the experimental fish used for swim performance tests and respirometry in swim-tunnels**.

**Group**	***N***	***BW* (g)**	***TL* (mm)**	***FL* (mm)**	***K***
1	12	34 ± 3	145 ± 4	128 ± 4	1.06 ± 0.02
2	12	206 ± 14	206 ± 7	222 ± 6	1.26 ± 0.02
3	9	392 ± 16	311 ± 5	273 ± 4	1.31 ± 0.06

*BW, body weight; TL, total length; FL, fork length. Significant differences existed in BW, TL and FL from one group to the next (P < 0.05)*.

#### Swim performance tests and respirometry

For the swimming experiments, two 127 L Blazka-type swim tunnels were used (van den Thillart et al., 2004). In each swim tunnel, water from the housing tank was circulating. A bypass with a galvanic oxygen electrode in a 4-channel respirometry system (DAQ-PAC-G4; Loligo Systems Aps, Tjele, Denmark) allowed registration of oxygen consumption. During respirometry, water in the swim-tunnels was recirculated and total oxygen content dropped due to the oxygen consumption of the swimming fish. A low rate of background (bacterial) respiration was always detected and subsequently subtracted from fish oxygen consumption. The percentual decline of oxygen content was used to directly calculate COT according to the formula:

(1) $$COT=\frac{△sat_{\left(t\right)}\cdot mg_{O_{2}}}{m\cdot △d}$$

where, △*sat*_(*t*)_ is the % decline in oxygen saturation during the measurement interval, *mg*_*O*_2__ is the amount of oxygen in mg per % saturation under the given conditions, *m* is the body mass of the fish in kg and △*d* is the covered distance in *m*. By equaling the first derivative of the polynomial function that described the relation between COT and the swimming speed *U* to zero, the *U*_opt_ was calculated.

Before each swimming experiment, two fish were individually introduced in a swim-tunnel and were allowed to acclimatize for 1 h which did not appear to be stressful for the fish as they were spontaneously swimming around at low speeds. Then, oxygen consumption was measured while swimming at consecutive speeds of 0.20, 0.40, 0.60, 0.80, and 1.00 m s^−1^ for 1 h per speed. At each speed, the fish were first allowed to acclimatize for 15 min before the oxygen content in the tunnels was measured for 30 min or until oxygen levels had dropped below 70% saturation. After each period of oxygen measurements the system was flushed to restore oxygen content to saturation levels. The experiment was terminated when either the fish fatigued (stopped swimming, hit the back fence and could not be stimulated to swim again) or when the fish had swum at 1.00 m s^−1^ for 1 h. Oxygen data from fish that stopped swimming at speeds <0.60 m s^−1^ were not used. Also data of some smaller fish that were observed to benefit from reduced flow by continuously swimming very close to the walls of the swim-tunnels at the highest speeds were excluded for those speeds. The oxygen consumption data were plotted against the swimming speeds in m s^−1^.

After swimming, each fish was anesthetized in clove oil (1:10 diluted in absolute ethanol and used as 2 mL in 10 L water). The anesthetized fish was measured for total length (TL) and fork length (FL).

### Swim training test in a swim-flume

#### The swim-flume, flow induction and turbulence

**Figure 1:**
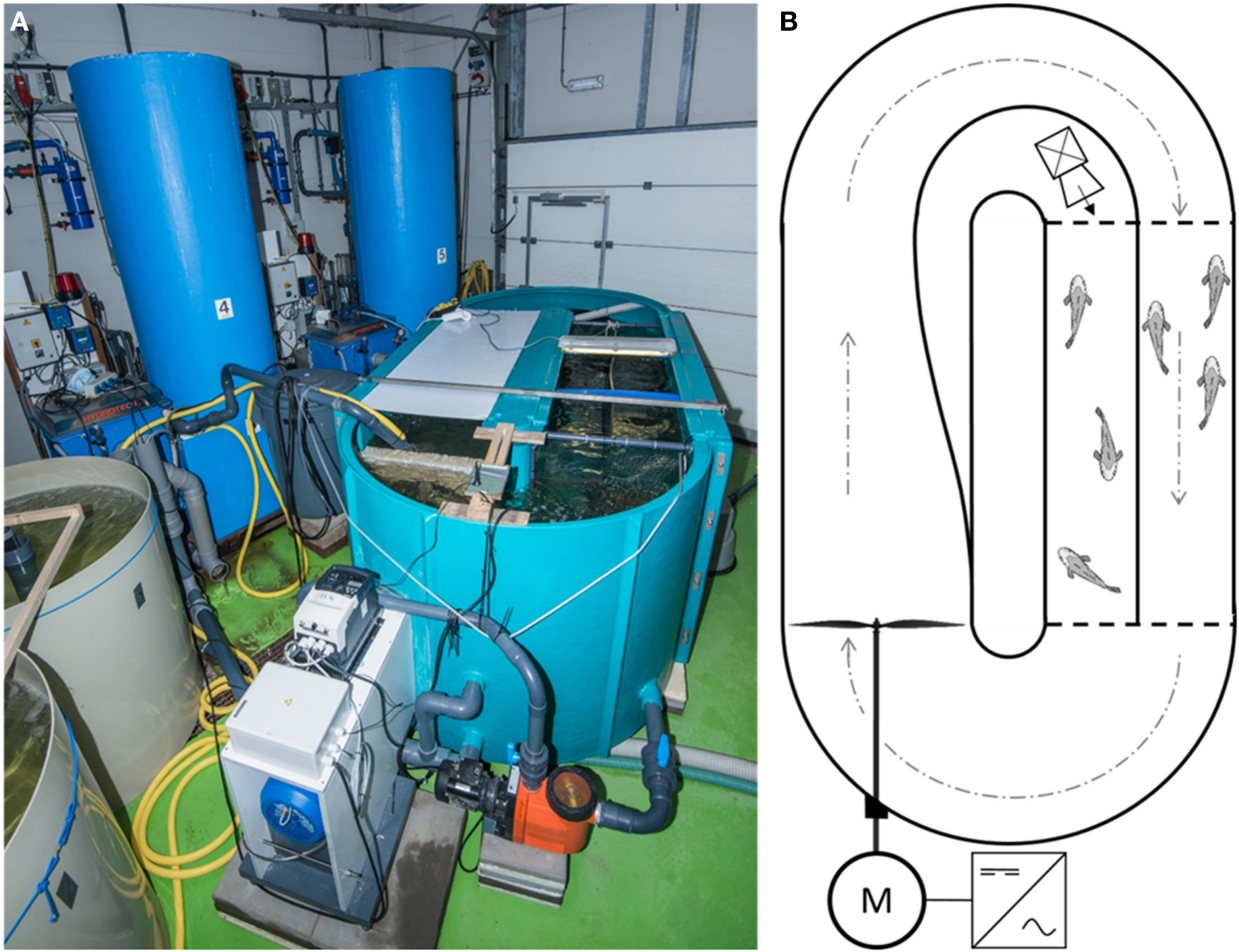
**The constructed swim-flume. (A)** side-view; and **(B)** schematic overview. An industrial inverter provided an alternating current with adjustable frequency (Hz) to an electric motor, which in turn powered the impeller. Water was led through the swimming compartment, forcing fish (*n* = 23) to swim at the optimal swimming speed. Fish (*n* = 23) in the rest-compartment were not subjected to water flow and displayed spontaneous activity. To maintain high water quality in the rest-compartment, low water flow was generated by a small pump, which was placed in the curve outside the compartment. See text for detailed description.

The experiment was conducted in a 3600 L oval-shaped Brett-type swim-flume (3.0 × 2.0 × 1.0 m; Figure 1; Brett, 1964) placed in a climatized room. The whole water volume of the recirculating system was pumped in 1 h over a filter system consisting of a Hydrotech drum filter (model HDF 501-1P, Hydrotech AB, Vellinge, Sweden), a trickle filter (Fleuren & Nooijen BV, Nederweert, the Netherlands), a 200 L biological moving bed biofilm reactor (MBBR) and a protein skimmer (Sander Aquarientechnik, Uetze-Eltze, Germany), all connected to a 400 L sump. Additionally, water from the 400 L sump was continuously pumped over a UV-filter (Proclear UV30 Advantage, Tropical Marine Centre Ltd. Hertfordshire, UK) and a heat exchanger (Maxicool XGL18HDA, Maxicool BV, Wessem, the Netherlands, modified by Climate4 u.nl, Valkenswaard, the Netherlands) to maintain water quality and water temperature, respectively. In one of the straight ends of the flume, two mesh fences (green polyester coated steel, 11 mm mesh size) were used to construct a compartment of 200 × 70 cm (Figure 1C). This compartment was divided by a PVC sheet (10 mm thick), thereby creating two 525 L sub-compartments, each measuring 200 cm (“x”) × 35 cm (“y”) × 70 cm (“z”) (length × width × depth). The resulting inner sub-compartment, where water flow was nil, was used to house the resting fish. The outer compartment, where water flow was maximal, was used to house the swimming fish. Ample water circulation to maintain water quality in the inner compartment was ensured by installing a pump (Aqua Ocean Runner OR 6500, Aqua Medic, Loveland, CO, USA) in the curve that flushed water through the compartment at a rate of 6500 l h^−1^.

Water flow was generated at the start of the straight end opposite the two compartments by an impeller connected to an electric motor (KLEEdrive MS2 132M-4 B3 (7.5 kW), Brd. Klee A/S, Albertslund, Denmark; Figure 1). The motor was powered by an industrial inverter (IP66, model no. BFI-E2-34-0180-3F4#, Beijer Electronics, Malmö, Sweden) with an adjustable alternating current (AC) output frequency between 0 and 50 Hz. This set-up to generate water flow was designed and first tested by Loligo Systems ApS (Tjele, Denmark). Additionally, a Speck pump (Badu 90/13, 0.55 kW with a capacity of 13 m^3^ h^−1^; Speck Pumps, Jacksonville, USA) continuously generated water circulation throughout the flume to safeguard water quality when the impeller was stationary.

Prior to the start of the experiment, water velocity in the swim compartment was measured using a downward-faced Vectrino acoustic Doppler velocimeter (ADV; Nortek AS, Rud, Norway) with its focal point at the center of the water column, 25 cm from the upstream fence. When the impeller was stationary, flow in the swimming compartment was measured. Inverter frequencies were subsequently increased with 0.5 Hz increments from 2.5 to 8.5 Hz, and after each increment, water flow was left to stabilize for 5 min, after which water velocities were measured in three dimensions (velocities *u*, *v*, and *w* in directions x, y, and z, respectively, as described above) for 10 s with a sampling rate of 10 Hz.

To quantify turbulence throughout the compartment, the inverter was set to 7.0 Hz, corresponding to a mean water velocity in the swimming compartment of 79 cm s^−1^, after which water velocities were measured within the swimming compartment at three horizontal transects (25, 100, and 175 cm), three vertical transects (8, 17.5, and 27 cm) at three depths (15, 37, and 55 cm) which resulted in a total of 27 measurements. Using velocities *u*, *v*, and *w*, the dimensionless turbulence intensity (TI) was calculated according to Liao and Cotel (2013) at each of the 27 locations:

(2) $$TI=u^{\prime }/{\left({\bar{u}}^{2}+{\bar{v}}^{2}+{\bar{w}}^{2}\right)}^{1/2}$$

where *u*′ is the standard deviation of velocity *u* and *u*, *v* and *w* are the average velocities in direction x, y and z, respectively.

#### Experimental fish and conditions

Juvenile yellowtail kingfish (*n* = 56) from the farm Silt BV (IJmuiden, the Netherlands) were randomly assigned to the rest-compartment (“REST”; *n* = 28) or the swimming compartment (“SWIM”; *n* = 28) of the flume and were acclimatized for 2 days.

After acclimatization, fish were not fed for 24 h and then ten fish (five from each compartment) were randomly selected and sampled (as “START” group). Individual fish were anesthetized in clove oil and total length (TL in mm) and body weight (BW in g) were measured from which Fulton's condition factor K was calculated, after which fish were euthanized by decapitation. The heart was dissected and heart weight (HW) was determined.

One day after the START group was sampled, the swimming trial commenced and the swimmers (*n* = 23) were forced to swim at *U*_opt_ in a sustained manner while resters (*n* = 23) were allowed to perform spontaneous activity at low water flow. Fish were fed commercial feed (Efico Sigma 570 No 6.5, Biomar A/S, Brande, Denmark), three times per day on weekdays and twice per day during weekends. The water flow was stopped during feeding sessions. First, the resters were fed until apparent satiation and the amount of feed given was determined. Then, swimmers were pair-fed and received the same amount as the resters. After feeding, fish were left for 15 min and then the water velocity was gradually increased to *U*_opt_. Fish were fed 2.65 ± 0.13 % BW d^−1^ and food conversion ratio (FCR) for both treatments was calculated as the ratio of biomass gain (wet weight in g) to total food intake (g).

Fish were checked for swimming behavior at least twice per day. Experimental fish did not show any visible signs of stress or fatigue, nor did infections, disease, or mortality occur. During the experiment, salinity was maintained at 26.2 ± 0.2‰ by mixing natural seawater (from the estuary Oosterschelde) with tap water. Mean water temperature was maintained at 23.6 ± 0.1°C. Oxygen gas was supplied through a ceramic diffuser downstream of the two compartments. Hundred percentage system volume was replaced daily to ensure high water quality. Water quality was monitored daily for O_2_, pH, NH_4_ and NO_2_, while NO_3_ was checked seven times throughout the 18-day experimental period. Oxygen levels were 7.67 ± 0.07 mg L^−1^, pH was 7.42 ± 0.029, NH_4_-N averaged 0.8 ± 0.2 mg L^−1^ (min 0 mg L^−1^ and max 2.3 mg L^−1^), NO_2_-N averaged 1.4 ± 0.2 mg L^−1^ (min 0.2 mg L^−1^ and max 3.1 mg L^−1^) and NO_3_-N averaged 14.8 ± 3.5 mg L^−1^ (min 2.01 mg L^−1^, measured after replacing one system volume, max 19.5 mg L^−1^, measured after 16 h without any water replacement) over 21 days. Nitrogenous waste-products were within safe limits for yellowtail aquaculture (Pierce et al., 1993; Colt, 2006).

After the 18-day experimental period, fish were not fed for 24 h, after which ten fish per treatment were collected and sampled as described for the START group. Of the remaining fish (*n* = 13 per treatment), blood flow was determined by Doppler ultrasound imaging, using an Esaote MyLabFive Vet ultrasonography unit (Esaote Europe BV, Maastricht, the Netherlands) with a 18 MHz LA 435 ultrasound transducer (Easote). Blood flow was visualized using the brightness (B) mode, Colour Flow Mapping (CFM) and Pulse Wave (PW) with the following settings: Velocity 89%; Angle (θ) + 60°; Depth 6 cm, Gain,_B_ 76%, Gain,_CFM_ 70%, Gain,_PW_ 52%; Frequency,_B_ 18 MHz, Frequency_CFM_, 8 MHz, and Frequency,_PW_ 8 MHz. Anesthetized fish were positioned on a table with the right lateral side facing upwards. The ultrasound transducer was covered with Aquasonic ultrasound transmission gel (Parker Laboratories Inc., Fairfield, NJ, USA) and held motionless against the ventral side of the fish near the head, in a longitudinal direction, allowing visualization of blood flow in the ventral artery, just downstream of the *bulbus arteriosus* (Figure S1). The scanning procedure was completed within 2 min. Blood flow was determined using the “El-Flow” function of the ADV. Using this function, the user manually traces the contour of the blood velocity graph and the width of the blood vessel is indicated. The ADV subsequently calculates the time average velocity as well as the cross-sectional area of the vessel, assuming a circular shape. Using these two parameters, the ADV calculates blood flow in mL min^−1^. After ultrasonography, fish were measured for TL and BW, after which fish were euthanized by decapitation.

Size measurements were used to calculate weight-specific growth rates for both swimmers and resters:

(3) $$SGR_{w}=\left(\ln\left(W_{f}\right)-\ln\left(W_{i}\right)\right)\times \frac{100}{t}$$

where *W_f_* is the final average weight of either resters or swimmers (g), *W_i_* is the initial average weight of the START group (g) and *t* is time between measurements (d).

### Statistics

BW, TL, and FL data in part I (Swim performance tests and respirometry in swim-tunnels) were normally distributed and tested for differences occurring between the three size classes (ANOVA, *P* < 0.05). K data were tested with Kruskal–Wallis tests.

All data in part II (Swim training test in a swim-flume) showed normal distribution (Shapiro–Wilk tests). To test for background effects over time, resters were compared to the START group. To test for treatment effects, swimmers were compared to resters. TL and BW were compared using student's *t*-tests with one-tailed probabilities, while K was compared using Mann–Whitney *U*-tests with two-tailed probabilities. Analysis of covariance (ANCOVA) with BW as the cofactor was performed on log transformed unpaired observations in search for group effects in the parameters HW and BF. Parameters were compared between resters and the START group and between swimmers and resters. In case there was no significant effect of the cofactor BW, ANOVA was used to determine whether there was a group effect.

Differences with *P* < 0.05 were considered significant. All data are presented as mean ± standard error (SE).

## Results

### Swimming performance tests and respirometry in swim-tunnels

#### Size

Size parameters (BW, TL, FL, and K) significantly increased from one experimental group to the next (*P* < 0.05; Table 1).

#### Swimming behavior

Seven fish of the smallest size group 1 at speeds of 0.80 m s^−1^ benefited from reduced drag by continuously swimming close to the walls of the swimming tunnels. One fish of group 1, two of group 2 and one of group 3 could not swim and appeared stressed in the swim-tunnels. Three fish of group 1 (critical swimming speed *U*_crit_ = 0.30 ± 0.06 m s^−1^ or 2.16 ± 0.47 BL s^−1^; calculated like Brett, 1964), three fish of group 2 (*U*_crit_ = 0.71 ± 0.15 m s^−1^ or 2.61 ± 0.53 BL s^−1^) and one fish of group 3 (*U*_crit_ = 0.60 m s^−1^ or 1.88 BL s^−1^) fatigued before swimming 1 h at 1 m s^−1^. One fish of group 1 was able to swim 1 h at 1 m s^−1^ (equal to 6.21 BL s^−1^) without swimming close to the walls, as well as seven of group 2 (represents 58% of the fish; equal to 4.12 ± 0.15 BL s^−1^) and seven of group 3 (represents 78% of the fish; equal to 3.21 ± 0.06 BL s^−1^).

#### Cost of transport (COT) and optimal swimming speeds (U_opt_)

**Figure 2:**
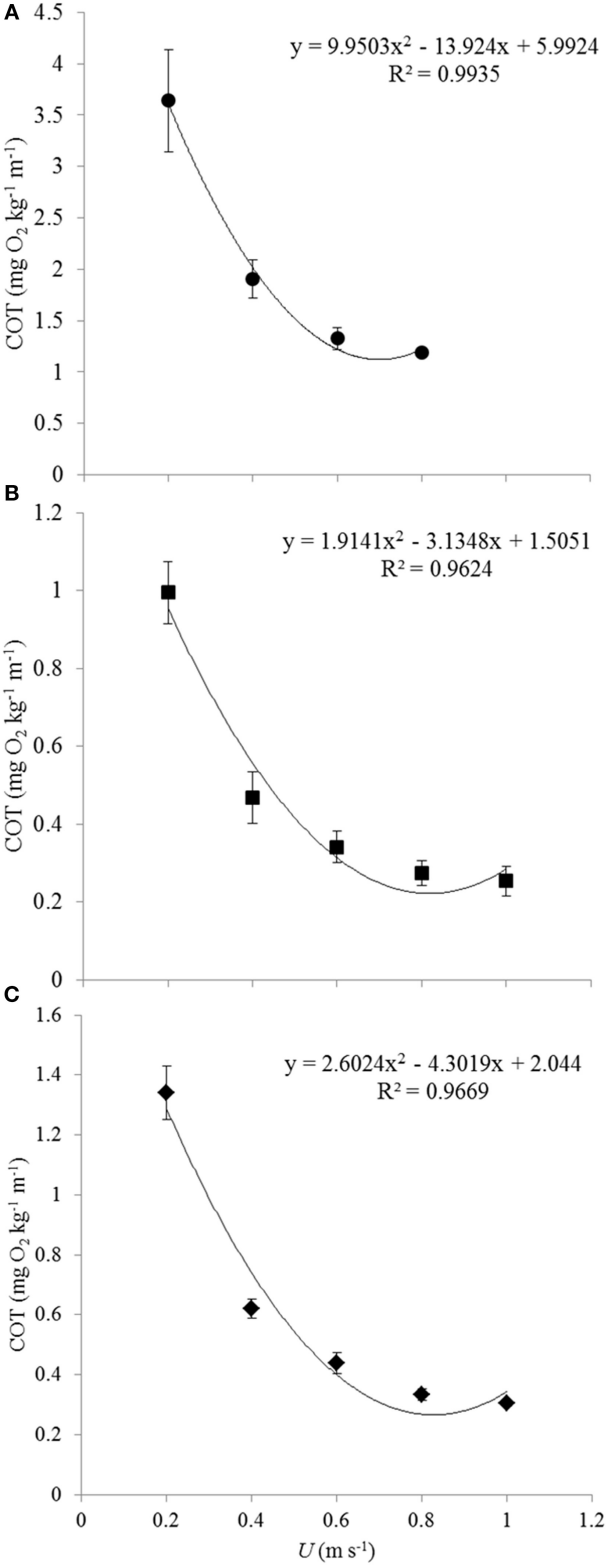
**Average COT values for each of the three size groups and polynomial plotting of trend-lines**. Each of the graphs representing the subsequent size groups gives the polynomial relation between COT and *U* and the *R*^2^-value. Averages and standard errors are based on data of decreasing numbers of fish for increasing speeds because of fish fatiguing and because of rejecting data of fish that were utilizing the lower flows near the wall of the swim-tunnel: **(A)** 0.20 m s^−1^: *n* = 7; 0.40 m s^−1^: *n* = 7; 0.60 m s^−1^: *n* = 6; 0.80 m s^−1^: *n* = 2, **(B)** 0.20 m s^−1^: *n* = 5; 0.40 m s^−1^: *n* = 8; 0.60 m s^−1^: *n* = 8; 0.80 m s^−1^: *n* = 7; 1.00 m s^−1^: *n* = 6, **(C)** 0.20 m s^−1^: *n* = 7; 0.40 m s^−1^: *n* = 7; 0.60 m s^−1^: *n* = 7; 0.80 m s^−1^: *n* = 6; 1.00 m s^−1^: *n* = 5. COT values of the smaller fish of group 1 were generally much higher than those of groups 2 and 3. Individual variation in COT decreased at increasing swimming speeds.

The calculated cost of transport (COT) values were plotted against the swimming velocities as polynomial *U*-shaped curves for each of the subsequent groups (Figures 2A–C). Only in case of the smallest size group 1 the polynomial curve was actually the best fitting (*R*^2^ = 0.9935). For groups 2 and 3, most of the fish were still swimming at 1 m s^−1^ so that the minimal COT value determining *U*_opt_ may not have been reached. Polynomial curves (for groups 2 and 3, respectively, *R*^2^-values of 0.9624 and 0.9669) were plotted to calculate *U*_opt_-values for both groups. The average COT was higher in group 1 as compared to the fish of groups 2 and 3 (Figure 2). The polynomial for group 1 followed the equation *y* = 9.9503x^2^ − 13.924x + 5.9924; for group 2 the equation was *y* = 1.9141x^2^ − 3.1348x + 1.5051; and for group 3 the equation was *y* = 2.6024x^2^ − 4.3019x + 2.044 (Table 2). The absolute *U*_opt_ (in m s^−1^) increased for each of the subsequent size groups from 0.70 m s^−1^ for group 1; to 0.82 m s^−1^ for group 2; to 0.85 m s^−1^ for group 3 (Table 2). The relative *U*_opt_ (in BL s^−1^) decreased: 4.83 BL s^−1^ for group 1, 3.25 BL s^−1^ for group 2, and 2.73 BL s^−1^ for group 3 (Table 2).

**Table 2:** **Optimal swimming speeds (*U*_opt_) per group in m s^−1^ and BL s^−1^**.

**Group**	***y(x)***	***y′(x)***	***U*_opt_ (m s^−1^)**	***U*_opt_ (BL s^−1^)**
1	*y* = 9.9503*x*^2^ − 13.924*x* + 5.9924	19.9006*x* − 13.924 = 0	0.70	4.83
2	*y* = 1.9141*x*^2^ − 3.1348*x* + 1.5051	3.8282*x* − 3.1348 = 0	0.82	3.25
3	*y* = 2.6024*x*^2^ − 4.3019*x* + 2.044	5.2048*x* − 4.3019 = 0	0.85	2.73

*Polynomial equations are shown for COT vs. U and their first derivatives. U_opt_ is defined as the speed with lowest COT and calculated by equaling the first derivative of each of these equations to zero. U_opt_ is given in absolute terms in m s^−1^ and in relative terms as BL s^−1^. The absolute U_opt_-values increase while the relative U_opt_-values decrease at increasing size*.

### Swim training test in a swim-flume

#### Flow and turbulence

Once the impeller was stationary, flow in the swimming compartment was 8.06 ± 0.27 cm s^−1^ as a result of the circulation generated by the Speck pump. Horizontal water velocity (*u*) in the swimming compartment increased proportionally to the AC-frequency of the inverter (*R*^2^ = 0.9977; Figure S2). Turbulence intensity (TI) in the swim compartment was subsequently calculated as the average over the 27 measurements. At a mean water velocity of 79 cm s^−1^ in the swimming compartment, TI was 0.083 ± 0.0032.

#### Swimming behavior

Swimmers displayed rheotactic behavior, grouping together in a school near the upstream fence of the compartment. The flow created by the impeller forced these fish to swim sustainably. Fish in the resting compartment displayed spontaneous swimming activity. After 18 days of continuous swimming, swimmers had swam an equivalent distance of 1250 km.

#### Fish growth

**Figure 3:**
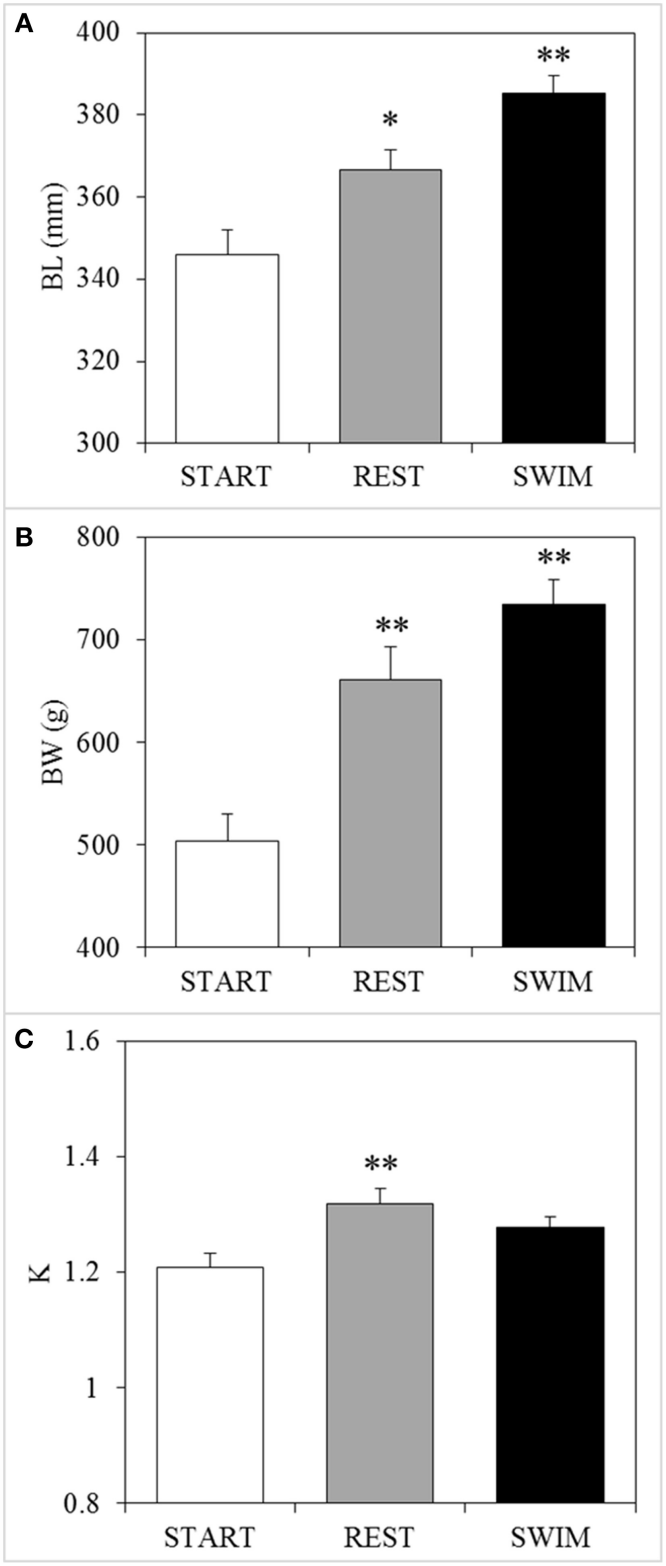
**Swimming-enhanced growth**. After 18 days, swimmers (“SWIM,” *n* = 23) showed **(A)** a significant 92% greater increase in body length (BL) and **(B)** a significant 46% greater increase in body weight (BW) as compared to resters (“REST,” *n* = 23), while **(C)** Fulton's condition factor (K) was not different between the two groups. Asterisks above REST-bars indicate a significant difference (^*^P < 0.05; ^**^P < 0.01) between the REST group and the START-group (*n* = 10: “time”-effects), while asterisks above SWIM-bars indicate significant differences between swimmers and resters (*n* = 23: “treatment”-effects).

Mean TL of the START group was 346 ± 6 mm and *U*_opt_ for this size class of fish was determined at 2.46 BL s^−1^ (water velocity of 0.85 m s^−1^). After 18 days at *U*_opt_ speeds, swimmers had grown an average 39 mm to 385 ± 4 mm whilst resters only grew 21 mm to 367 ± 5 mm; a 92% greater increase in length (*P* < 0.05; Figure 3). Furthermore, swimmers (735 ± 23 g) increased 231 g in BW as compared to the START group (504 ± 27 g), which is a 46% greater increase than the resters (661 ± 32 g) (*P* = 0.035; Figure 3). SGR was 40% higher for swimmers (2.1% BW d^−1^) as compared to resters (1.5% BW d^−1^). Swimmers and resters had similar condition factors (*K* = 1.28 and 1.32, respectively) (*P* > 0.05; Figure 3). Feed conversion ratio was lower and thus more efficient at 1.21 compared to 1.77 in resters.

#### Heart and blood

HW (with BW as cofactor) showed any significant differences between groups (Table 3). Swimmers had a blood flow of 44 ± 3 mL min^−1^, resters 34 ± 3 mL min^−1^ (Table 3). Swimmers thus showed a significantly higher (+31%) cardiac output in the ventral artery (*P* = 0.026; Table 3).

**Table 3:** **Effects of performing sustained exercise for 18 days on heart weight and blood flow**.

**Parameters**	**START**	**REST**	**SWIM**
*HW* (g)	0.95 ± 0.05	1.21 ± 0.06	1.22 ± 0.06
*BF* (mL min^−1^)		34 ± 3	**44 ± 3**

*The START group (n = 10; TL = 346 ± 6 mm, BW = 504 ± 27 g) was sampled before the swim trial commenced. Fish swam (“SWIM,” n = 23; TL = 385 ± 4 mm, BW = 735 ± 23 g) at 2.46 BL s^−1^ or were allowed to perform spontaneous movement (“REST,” n = 23; TL = 367 ± 4 mm, BW = 661 ± 32 g) for 18 days. Hearts of 10 fish per group were weighted (HW). Blood flow in the ventral artery (BF) was measured in 13 resters and 13 swimmers. Parameters were statistically tested. Swimmers had a significant (P < 0.05) higher blood flow than resters, detected using pairwise ANCOVA with BW as cofactor and indicated in bold*.

## Discussion

In this study we have established optimal swimming speeds for juvenile yellowtail kingfish allowing to apply this knowledge for an experimental swim-training trial of 18 days to investigate the effects of swimming exercise on growth performance and cardiac output. Exercise-enhanced growth was robust and not caused by increased feed intake. It may have been caused by increased feeding efficiency indicated by a lower FCR. Doppler ultrasound imaging showed a cardiac output which was significantly higher in exercised fish, no such data have been shown for fish before (to the best of our knowledge).

**Figure 4:**
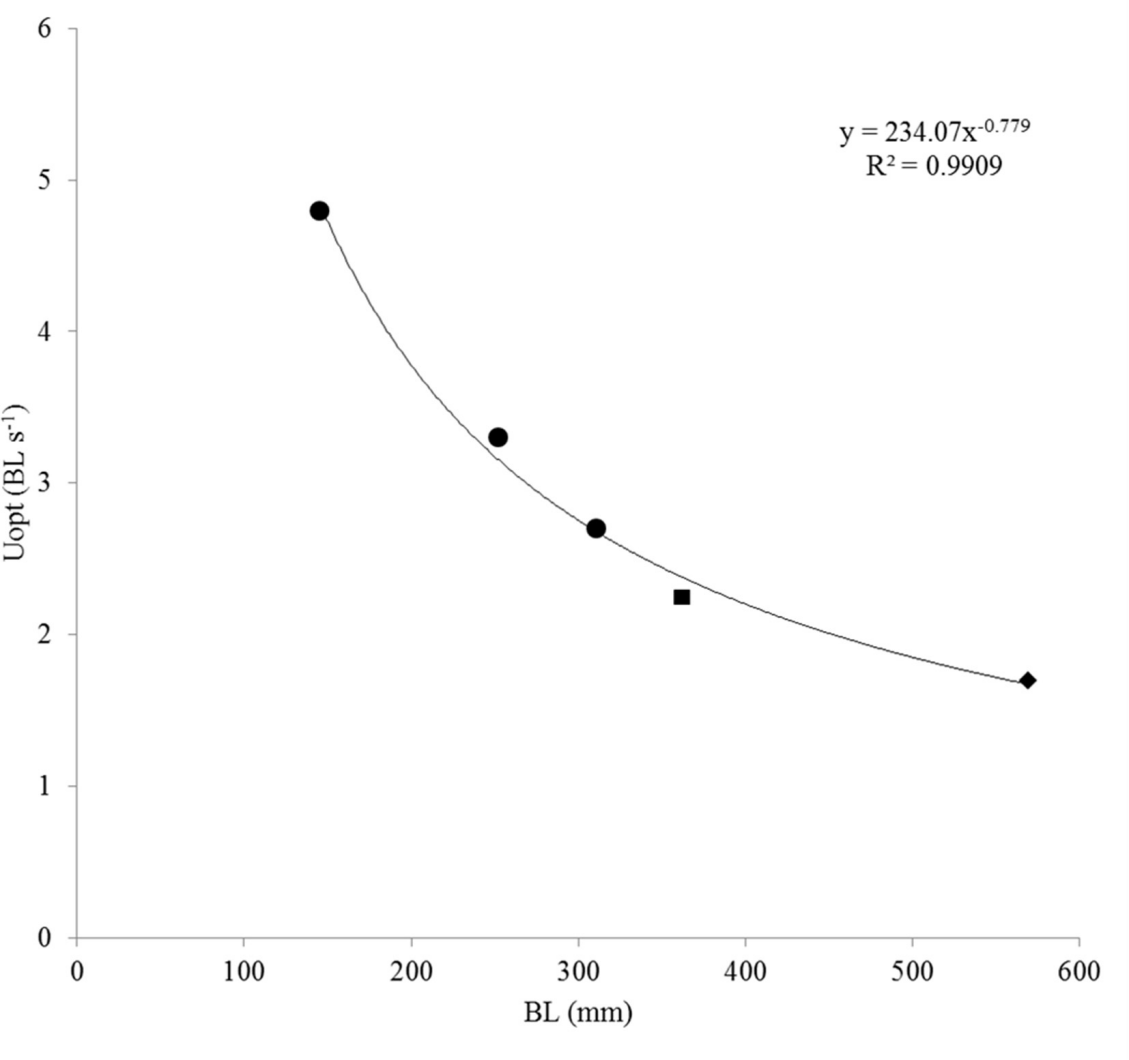
**Optimal swimming speed *U*_opt_ (in body length per second BL s^−1^) in relation to size in BL**. Shown are the three *U*_opt_-values obtained in this study (circles) of group 1 (4.85 BL s^−1^), group 2 (3.25 BL s^−1^) and group 3 (2.73 BL s^−1^); the *U*_opt_ (2.25 BL s^−1^) of yellowtail kingfish of size 362 ± 15 mm, 699 ± 39 g as reported by Brown et al. (2011; square) and the *U*_opt_ (1.7 BL s^−1^) of yellowtail kingfish of size 569 ± 26 mm, 2.35 ± 0.31 kg as reported by Clark and Seymour (2006; diamond). Best of fit is a power function: *y* = 234.07x^−0.779^ (*R*^2^ = 0.9909). Note that BL for fish of the study by Brown et al. (2011; square) is in fork length, adding 30–40 mm to estimate the TL-values leads to a perfect fit of this data point on the trend line.

Plotting polynomial curves for each of the three size classes enabled us to calculate the swimming speeds at which COT was minimal and that correspond to the *U*_opt_ (Palstra et al., 2008). Calculating these speeds revealed increasing absolute *U*_opt_-values in m s^−1^ with increasing body length which were decreasing when expressed relative to body length (Table 2). As most of the larger fish were still swimming at 1 m s^−1^, polynomial curves predicted by the classical exponential relation between *U* and MO_2_ (Jones and Randall, 1978), were not the best fitting curves for groups 2 and 3. Values were however well in line with the two *U*_opt_-values known from literature for larger *S. lalandi*: 2.25 BL s^−1^ for yellowtails of 362 mm BL (Brown et al., 2011) and 1.7 BL s^−1^ for yellowtails of 569 mm BL (Clark and Seymour, 2006). By plotting the three *U*_opt_-values for juveniles of this study with these two *U*_opt_-values for larger fish, a relation can be established of *U*_opt_ (BL s^−1^) = 234.07(BL)^−0.779^ (*R*^2^ = 0.9909; Figure 4). This formula describes the relation between body length and optimal swimming speed over a size range of 145–569 mm BL and therewith provides a tool to calculate *U*_opt_-values for fish in this size range.

Eighteen days of sustained exercise training at optimal swimming speed enhanced growth of juvenile yellowtail kingfish substantially with a 92% gain in length and a 46% gain in body weight as compared to the increase seen in controls. Because resters and swimmers were housed in the same flume under the same conditions and given equal feed rations, increased SGR*_w_* in swimmers is predicted to be a result of sustained swimming exercise alone. Our results support a growing body of work that shows growth-stimulating effects of sustained exercise in active metabolic fish, mainly salmonids and pelagic teleosts (reviewed by Davison and Herbert, 2013). Typically, the enhancement of growth performance (e.g., increase in body weight) by exercise is ~40% in these species (Davison and Herbert, 2013). Brown et al. (2011) found exercise-enhanced growth performance in *S. lalandi* but only a moderate 10% increase in SGR*_w_*. This result is contrary to the results of Yogata and Oku (2000) who found a 36–38% weight gain in exercised fingerling *S. quinqueradiata* which better fits the expectation. Brown et al. (2011) speculated on the difference in growth stimulation comparing their study with the study of Yogata and Oku (2000), e.g., a suboptimal temperature (21.1 vs. 22.0–24.6°C, respectively) and larger size of the fish (1600 vs. 4 g, respectively). The most appropriate explanation may actually be the lower applied flow: 0.75 BL s^−1^ vs. 1.0–2.25 BL s^−1^, respectively, although body size and temperature may also affect the relationship between *U*_opt_ and growth in *Seriola* sp. According to the functional relation that we found, the *U*_opt_ for the fish used by Brown et al. (2011; 476 mm BL) would be 1.92 BL s^−1^ and thus was the reported speed for optimal growth, suboptimal in respirometric terms (although routine speed was not recorded, estimated to be slightly lower at ~0.5–0.75 BL s^−1^, and calculated to correspond to 1.9–2.4 BL s^−1^ when swimming in a straight line which would correspond to the *U*_opt_ that can be calculated with the functional relation that we found). We believe that our study shows exercise-enhanced growth for *Seriola* spp. of 46% because fish were forced to swim at their *optimal* swimming speed. This also agrees with the deduced highest weight gain in fingerling *S. quinqueradiata* as reported by Yogata and Oku (2000; ~45% in Figure 1). From our functional relation we can however not determine what would be *U*_opt_ for fish <145 mm and thus we do not know which would be *U*_opt_ for the fingerlings used by Yogata and Oku (2000). The reason why Brown et al. (2011) did not find more pronounced growth stimulation at the higher applied swimming speeds of 1.5 BL s^−1^, and perhaps also at 2.25 BL s^−1^, remains however a mystery according to the hypothesis that growth stimulation would occur at *U*_opt_. Our findings may support this hypothesis.

Sustained exercise has been demonstrated to improve food conversion rates (FCRs) in several active teleosts (Jobling et al., 1993; Davison, 1997; Magnoni et al., 2013). The present study revealed a 32% lower FCR for swimmers as compared to resters, supporting previous studies on *S. lalandi* (Brown et al., 2011) and *Seriola quinqueradiata* (Yogata and Oku, 2000). The mechanisms driving reduced FCRs in exercised fish are not yet fully scrutinized, but recent studies suggest that exercise increases nutrient uptake efficiency. For example, exercise increased carbohydrate turnover and promoted protein-uptake in white muscle in gilthead sea bream *S. aurata* (Felip et al., 2013) and in Atlantic salmon *Salmo salar*, higher energy efficiency and amino acid synthesis were detected in exercised smolts as compared to resting fish (Grisdale-Helland et al., 2013). Although our understanding of improved FCRs under exercise regimes is still limited, our results indicate that the implementation of sustained exercise in aquaculture has the potential to extensively improve growth performance and reduce feeding costs at the same time.

Heart weights did not increase as a treatment effect but what we did find was a significantly increased blood flow in the ventral artery in exercised fish, just after the *bulbus arteriosus*, reflecting a higher cardiac output. Although aerobic exercise has been shown to increase cardiac growth in some teleosts, effects are often small and variable (reviewed by Gamperl and Farrell, 2004). *S. lalandi* has previously been shown to increase cardiac output by exercise training, without increasing cardiac stroke volume but by increasing heart rate (Clark and Seymour, 2006), which supports a higher blood flow without the increase of HW as found in the current study. A similar observation was made in rainbow trout, where daily exercise cycles did not increase heart size (Farrell et al., 1990) but did increase heart capacity in exercised fish (Farrell et al., 1991).

Ultrasound imaging is extensively used for non-invasive gonadal observations and sex identification in teleost fishes (reviewed by Novelo and Tiersch, 2012). Currently, teleost blood flow is often determined by surgical insertion of Doppler flow probes, which requires deep anaesthetization of the animal, invasive surgical procedures with risks of infection, and leaves the fish with leads protruding from the body, which have to be connected to velocimeters (e.g., Thorarensen et al., 1993; Clark and Seymour, 2006; Petersen et al., 2011). When standardized for weight, swimmers in this experiment showed an average blood flow of 59 mL min^−1^ kg^−1^, which approximates the results of Clark and Seymour (2006), who measured a blood flow of 50 mL min^−1^ kg^−1^ in yellowtails using silastic Doppler flow cuffs. This similarity between the blood flows obtained by an ADV vs. flow cuffs supports the potential of ADVs as alternative method for blood flow measurement, although more comparative studies need to be performed to fully assess the ADV's potential. The use of ultrasound techniques in teleosts is advancing rapidly: recently Guitreau et al. (2012) used ultrasound imaging to visualize the ovaries of submersed, non-anesthetized, unrestrained catfish. If protocols are optimized to allow the use of ADVs on non-anesthetized, submerged fish, e.g., by using water as a ultrasound transducer medium, the use of ADVs might pose a non-invasive, low-stress alternative to currently used surgical methods.

Vectrino measurements along the x-, y-, and z-axis in the newly designed swim-flume with motor-driven impeller showed a rather uniform flow in the swimming compartment in the applied configuration. When uniformity in flow is lacking, turbulence may have serious impact on the energy expenditure of swimming fish (Liao and Cotel, 2013). For example, fish swimming in vortices downstream of bluff bodies showed reduced muscle activity (Liao et al., 2013), while irregular turbulence has been shown to be detrimental to swimming fish, leading to increased energy expenditure on stability requirements in complex flows (Lupandin et al., 2000; Enders et al., 2003; Liao and Cotel, 2013). In our study, turbulence intensity in the swimming compartment of the flume was 0.08, which is considered low (Lupandin et al., 2000; Pavlov and Skorobogatov, 2009), and should therefore not disturb the optimal swimming economy of the fish in the experimental setup.

The created flow forced fish to swim continuously and in a sustainable way. Fish in the resting compartment showed spontaneous swimming activity, like being performed in tanks at the farm. Vectrino flow measurements in three tanks on the farm showed that fish of 600–1800 g were subjected to flows of 0.19–0.34 m s^−1^, corresponding to ~0.62–0.86 BL s^−1^. Fish swam generally slightly faster than the flow indicating that the preferred swimming speeds were higher. Still, the preferred swimming speeds for the yellowtails under these conditions were not much higher than 1 BL s^−1^ and did by far not approach the optimal swimming speeds, contrary to a study on brook charr using a tilted raceway where preferred and optimal swimming speeds were found similar (Tudorache et al., 2011).

## Conclusions

Our study has delivered a formula to calculate optimal swimming speeds for juvenile yellowtail kingfish over a size range of 145–311 mm BL, and together with data from literature (Clark and Seymour, 2006; Brown et al., 2011) up to 569 mm BL: *U*_opt_ (BL s^−1^) = 234.07(BL)^−0.779^ (*R*^2^ = 0.9909). The formula was used to execute an experimental scale training experiment of 18 days. Results show that forced sustained exercise at optimal swimming speeds leads to a 92% greater increase in BL and 46% greater increase in BW, an exercise-induced growth stimulation that is much more pronounced than the 10% growth stimulation shown earlier for *S. lalandi* by Brown et al. (2011) and that is similar to the optimal growth stimulation for much smaller *S. quinqueradiata* juveniles (Yogata and Oku, 2000). The applied optimal swimming speed for the experimental fish in this study was 2.46 BL/s (0.85 m/s), much higher than the 0.75 BL s^−1^ at which Brown et al. (2011) found the highest growth rates, and also higher than the 1.5–2.0 BL s^−1^ as applied by Yogata and Oku (2000). Moreover, in this study non-swimming and swimming fish were given equal feeding rations (satiation for non-swimmers and restriction for swimmers) so that no differences in feed intake existed between both groups. The difference in growth is reflected by lower FCR for swimmers than for non-swimmers and is thus caused by higher feeding efficiency. It can be expected that when also swimmers are fed until satiation, exercise-enhanced growth will be even more pronounced. Exercise-enhanced growth is accompanied by higher blood flow in the ventral aorta, thus by increased cardiac output, a necessary adaptation in the aerobic phenotype to supply increased oxygen demands.

## Perspectives

The outcomes of this study on experimental scale will need to be validated in an industrial scale setting where several aspects may differ from the experimental situation (densities, feeding conditions, water quality parameters, etc.). One aspect that will be different is that not a swim-flume will be applied but tanks or perhaps raceways as part of RAS having very different hydraulics. It will be challenging to create the flows that are required to induce optimal swimming, especially for the larger sized fish (>500 mm). Flows could be created by using motor-driven impellers like in this study, pumps or gravity, but perhaps a good alternative may be a device that exploits the optomotor response to encourage exercise, like the Optoswim concept (Herbert et al., 2011; reviewed by Herbert, 2013). When validated, implementation of optimal swimming regimes in yellowtail aquaculture, particularly in on-land RAS, will lead to a much faster production cycle. Moreover, increased production by exercise will most probably be accompanied by improved health and welfare aspects.

## Author contributions

Conceived and designed the experiments: Arjan P. Palstra, Kees Kloet. Performed the experiments: Arjan P. Palstra, Daan Mes, Kasper Kusters, Jonathan A. C. Roques. Analyzed the data: Arjan P. Palstra, Daan Mes, Kasper Kusters, Jonathan A. C. Roques. Wrote the paper: Arjan P. Palstra, Daan Mes, Kasper Kusters, Gert Flik, Robbert J. W. Blonk.

### Conflict of interest statement

The authors declare that the research was conducted in the absence of any commercial or financial relationships that could be construed as a potential conflict of interest.
