# Supplementary material for: Forced sustained swimming exercise at optimal speed enhances growth of juvenile yellowtail kingfish (Seriola lalandi)
Source: Front Physiol. 2015 Jan 8;5:506. doi: 10.3389/fphys.2014.00506 (PMC4287099; doi:10.3389/fphys.2014.00506)
Supplement: Supplementary file 1 [file DataSheet1.DOCX]

***Supplementary Material***

**Forced sustained swimming exercise at optimal speed enhances growth of juvenile yellowtail kingfish (*Seriola lalandi*)**

**Arjan P. Palstra^1^*, Daan Mes^1^, Kasper Kusters^1^, Jonathan A.C. Roques^2^, Gert Flik^2^, Kees Kloet^3^, Robbert J.W. Blonk^1^**

^1^ Institute for Marine Resources and Ecosystem Studies (IMARES), Wageningen Aquaculture**, Wageningen University and Research Centre, Yerseke, The Netherlands

^2^ Department of Animal physiology, Institute for Water and Wetland Research, Radboud University Nijmegen, Nijmegen, The Netherlands

^3^ Silt BV, IJmuiden, The Netherlands

* Correspondence: Dr. Arjan P. Palstra, The Institute for Marine Resources and Ecosystem Studies (IMARES), Wageningen University and Research Centre, Korringaweg 5, 4401 NT Yerseke, The Netherlands.

[arjan.palstra@wur.nl](mailto:arjan.palstra@wur.nl)

1. **Supplementary Figures**


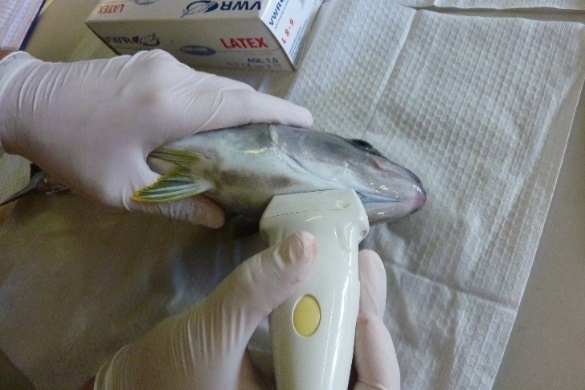


Supplementary Figure 1. The ultrasound transducer of the ADV was held in longitudinal direction along the ventral artery of the anaesthetized fish.


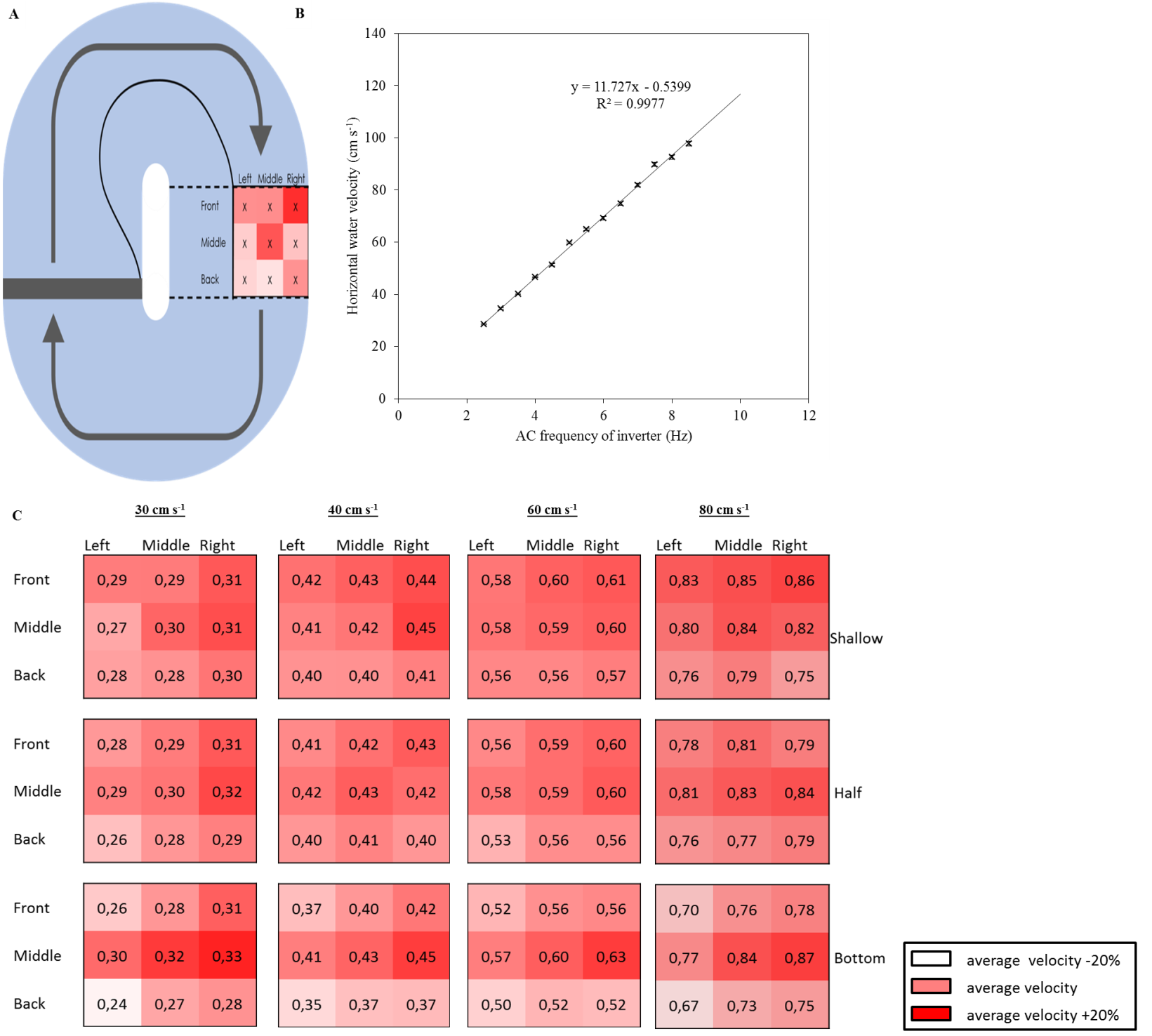


Supplementary Figure 2. Water velocities in the swim-flume measured using a Vectrino acoustic Doppler velocimeter (ADV). (A) The swim-flume, the swimming compartment and the 3*3 locations (*3 depths) where flow was measured; (B) Water velocities along the x-axis of the flow direction in the swimming compartment of the flume. Water velocities increased linearly with the alternate current (AC) frequency of the inverter which powered the electric motor. (C) Variation in velocities (white-to-red scale: see legend) at each of the 27 locations for four different speeds as determined from the linear relation in (B).
